# Inflammation proteomics datasets in the ALSPAC cohort

**DOI:** 10.12688/wellcomeopenres.18482.2

**Published:** 2024-02-06

**Authors:** Neil Goulding, Lucy J. Goudswaard, David A. Hughes, Laura J. Corbin, Alix Groom, Susan Ring, Nicholas J. Timpson, Abigail Fraser, Kate Northstone, Matthew Suderman

**Affiliations:** 1 Population Health Sciences, Bristol Medical School, University of Bristol, Bristol, UK; 2 Medical Research Council (MRC) Integrative Epidemiology Unit, University of Bristol, Bristol, UK

**Keywords:** Proteomics, Olink, inflammation, ALSPAC, birth cohort, inter-generational

## Abstract

Proteomics is the identification, detection and quantification of proteins within a biological sample. The complete set of proteins expressed by an organism is known as the proteome. The availability of new high-throughput proteomic technologies, such as Olink Proteomic Proximity Extension Assay (PEA) technology has enabled detailed investigation of the circulating proteome in large-scale epidemiological studies. In particular, the Olink® Target 96 inflammatory panel allows the measurement of 92 circulating inflammatory proteins. The Avon Longitudinal Study of Parents and Children (ALSPAC) is a prospective population-based cohort study which recruited pregnant women in 1991-1992 and has followed these women, their partners, and their offspring ever since. In this data note, we describe the newly-released proteomic data available in ALSPAC. Ninety-two proteins were analysed in 9000 blood plasma samples using the Olink® Target 96 inflammatory panel. Samples were derived from 2968 fasted mothers (mean age 47.5; Focus on Mothers 1 (FOM1)), 3005 non-fasted offspring at age 9 (Focus@9) and 3027 fasted offspring at age 24 (Focus@24). Post sample filtering, 1834 offspring have data at both timepoints and 1119 of those have data from their mother available. We performed quality control analyses using a standardised data processing workflow ( *metaboprep*) to produce a filtered dataset of 8983 samples for researchers to use in future analyses. Initial validation analyses indicate that IL-6 measured using the Olink® Target 96 inflammatory panel is highly correlated with IL-6 previously measured by clinical chemistry (Pearson’s correlation = 0.77) and we are able to reproduce the reported positive correlation between body mass index (BMI) and IL-6. The pre-processing and validation analyses indicate a rich proteomic dataset to further characterise the role of inflammation in health and disease.

## Introduction

Proteomics is a general term used to describe the identification, detection and quantification of proteins ^1^. At its most basic level, proteomics can be defined as ascertaining the identity and quantity of proteins in a tissue at any given timepoint. Protein identification can include the identification of protein isoforms and post-translational modifications which can be collectively referred to as protein quality or function, and quantity refers to measuring how much of each protein is in a sample. Both protein quality and quantity are influenced by genetic variation and environmental variation, and both can contribute to phenotypic variation and disease. Importantly, disease can be influenced by and can influence protein quantity ^2, 3^. Exploring proteome variation not only furthers our understanding of health and disease, it also aids in the identification of druggable targets and advances pharmacological interventions ^4^.

The standard methods for protein detection have historically included enzyme-linked immunosorbent assays (ELISAs) and Western blotting, however, these techniques are limited by the number of proteins that can be detected ^1^. More high-throughput methods include mass spectrometry, which can detect and quantify thousands of proteins. Yet, new proteomic technologies such as that developed by Olink Proteomics ^5^ (Uppsala, Sweden) enable more precise identification and quantification of a range of proteins at scale using sample volumes as low as 1 uL ^6^. The Olink platforms utilise proximity extension assay (PEA) technology, consisting of antibody pairs labeled with oligonucleotides, to measure proteins ^5, 7^. There are a range of panels (such as neurology, oncology, or inflammation) offered by Olink, each categorised by the function or associated disease classification(s) of included proteins.

Inflammation is the body's response to stimuli perceived to be harmful such as pathogens, damaged cells and toxins. Although inflammation is critical for health, chronic inflammation can be damaging and has been linked to the development and mediation of non-communicable diseases such as arthritis, diabetes, cancer and cardiovascular disease ^8^. Inflammation is therefore hypothesised to mediate the negative health effects of a variety of exposures associated with increased inflammation. For example, current evidence indicates that infection and inflammation in early life has important consequences for cardiovascular health later in life ^9^. Further, increased levels of inflammation have been reported in adults who had experienced adverse childhood experiences (ACEs) decades earlier ^10^ possibly explaining why these individuals also experience increased risk of non-communicable diseases. As such, comprehensive quantification of proteins associated with inflammation in a deeply phenotyped, multi-generational, longitudinal cohort may be used to further advance how inflammation influences disease outcomes.

Toward this aim, the Olink ^®^ Target 96 Inflammation panel was performed on 9000 samples from the Avon Longitudinal Study of Parents and Children (ALSPAC), which is a birth cohort in the former Avon area surrounding Bristol, UK ^11– 13^. Use of this cohort has made multiple contributions to the general understanding of disease risk factors, as well as mechanisms of disease. The detailed phenotypic measurements from early life in ALSPAC along with the quantification of 92 circulating inflammatory proteins measured by Olink provides a rich dataset to characterise the role of inflammation in health and in a vast range of disorders. The aims of this data note are to describe the collection and processing of Olink proteomic data in 9000 samples from 2968 mothers and 4198 offspring across two timepoints.

## Methods

### Overview of cohort

Pregnant women resident in the United Kingdom’s former county of Avon region surrounding Bristol, with expected dates of delivery 1st April 1991 to 31st December 1992, were invited to take part in the study. The initial number of pregnancies enrolled was 14,541 (for these at least one questionnaire had been returned or a “Children in Focus” clinic had been attended by 19/07/99). Of these initial pregnancies, there was a total of 14,676 fetuses, resulting in 14,062 live births and 13,988 children who were alive at 1 year of age.

When the oldest children were approximately 7 years of age, the initial sample was added to through the recruitment of eligible cases who had failed to join the study originally. As a result, when considering variables collected from the age of seven onwards (and potentially abstracted from obstetric notes) there are data available for more than the 14,541 pregnancies mentioned above. The number of new pregnancies not in the initial sample (known as Phase I enrolment) that are currently represented on the built files and reflecting enrolment status at the age of 24 is 913 (456, 262 and 195 recruited during Phases II, III and IV respectively), resulting in an additional 913 children being enrolled. The phases of enrolment are described in more detail in the cohort profile paper and its update. The total sample size for analyses using any data collected after the age of seven is therefore 15,454 pregnancies, resulting in 15,589 fetuses. Of these 14,901 were alive at 1 year of age.

Please note that the study website contains details of all the data that is available through a fully searchable data dictionary and variable search tool.

### Ethical approval

Ethical approval for the study was obtained from the ALSPAC Ethics and Law Committee and the Local Research Ethics Committees. Consent for biological samples has been collected in accordance with the Human Tissue Act (2004). Informed written consent for the use of data collected via questionnaires and clinics was obtained from participants following the recommendations of the ALSPAC Ethics and Law Committee at the time.

### Blood samples

**Figure 1.**
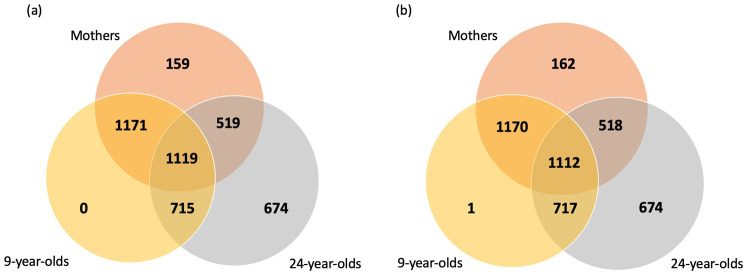
A Venn diagram of the longitudinal and family overlap in samples analysed by Olink. ( **a**) All 9000 ALSPAC samples sent to Olink and ( **b**) The 8983 samples in the filtered dataset.

Nine-thousand heparin-stored plasma samples were analysed by Olink, which were collected from three different age groups, namely mothers in midlife (n=2968) and offspring at approximate ages 9 (n=3005) and 24 (n=3027) years ( Table 1), with a large overlap across the three timepoints ( Figure 1). Blood was collected in lithium heparin tubes and placed on ice until processing. Samples were spun at 1300g for 10 minutes at 4–5°C. Plasma was aliquoted from the tube and stored at -80°C. Target time from sample collection until plasma frozen was 90 minutes ^14, 15^. The mothers and 24-year-olds fasted before the samples were taken, whereas the 9-year-olds did not. Due to funding and matching of families, it was not possible to analyse all samples from all timepoints. All samples which were sent to Olink were shipped on dry ice, then thawed and aliquoted into plates on arrival. Due to the technical challenges of sorting thousands of frozen samples, it was not possible to organise children’s repeated samples and mother-child pairs onto the same plates, and instead the order of samples on plates was randomised. 

**Table 1.** Subject characteristics of the study population. Characteristics are shown for the sample as a whole (N=8983) and split into mothers (N=2962), children (N=3000) and young adults (N=3021). Values of N are less for some of the characteristics due to missing data. Maternal/paternal information was used where data for offspring was not available. *BMI*, body mass index. * A-level = Advanced level qualification (optional examinations taken at the approximate age of 18). N/As indicate where it is not appropriate to provide a summary of the whole sample.

Characteristic	Whole sample	N	Mothers	N	Children	N	Young adults	N
**Age (years), mean (SD)**	27.2 (15.7)	8978	47.5 (4.4)	2958	9.9 (0.3)	3000	24.5 (0.8)	3020
**Female, n (%)**	6466 (72.0)	8983	2962 (100)	2962	1592 (53.1)	3000	1912 (63.3)	3021
**White ethnicity, n (%)**	7900 (96.6%)	8179	2675 (97.7)	2739	2655 (95.8)	2770	2570 (96.3)	2670
**BMI (kg/m ^2^), mean (SD)**	22.9 (5.9)	8898	26.4 (5.1)	2947	17.4 (2.6)	2962	24.7 (4.9)	2989
**Current smoker/exposed** ** to smoke, n (%)**	N/A	N/A	166 (8.5)	1957	664 (25.0)	2651	852 (28.6)	2978
Education above A-level *, **n (%)**	N/A	N/A	554 (20.2)	2748	Maternal: 583 (20.8)	2800	1086 (54.8)	1983
					Paternal: 730 (27.9)	2615		
**Manual occupation, n (%)**	N/A	N/A	132 (12.0)	1102	Maternal: 151 (13.4)	1124	Maternal: 126 (11.7)	1077
					Paternal: 677 (67.2)	1008	Paternal: 621 (66.4)	935

***Mothers***. The mothers’ samples that were analysed by Olink were collected from the 1 ^st^ Focus on Mothers clinic (FOM1) between December 2008 and December 2011 ^12^. In total, 4981 mothers attended the clinic, 4827 consented to have blood samples taken and 2968 (61%) of these samples were analysed by Olink.

***Focus@9.*** In total, 7719 children attended the Focus@9 clinic at approximately 9.5 years of age. Parents gave consent for 7236 children to have blood samples taken and 3005 (42%) of these samples were analysed by Olink.

***Focus@24***. In total, 4026 young persons attended the Focus@24 clinic at approximately 24 years of age, 3552 of which consented to having a blood sample taken and 3027 (85%) of these samples were analysed by Olink. 

**Figure 2.**
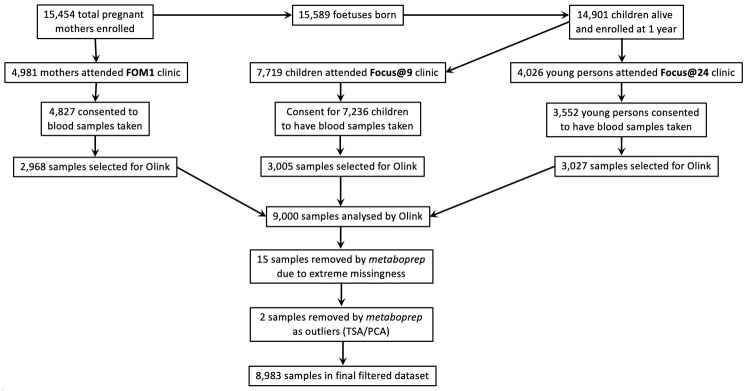
A flowchart indicating ALSPAC sample selection and *metaboprep* filtering steps for Olink samples. FOM: Focus on Mothers; TSA: total sum abundance; PCA: principal component analysis.

A flowchart for the selection of ALSPAC participants who had samples analysed by Olink is included in Figure 2.

***Longitudinal and family data*.** Across the 9000 mother and children/young person samples 2290 mothers have a child analysed at 9 years of age, 1638 at 24 years of age, and 1119 at both 9 and 24 years of age. In addition, 1834 of the children were analysed at both timepoints (9 and 24 years of age; see Figure 1a). All 9-year-olds were either analysed again at 24 years of age or their mother was also analysed.

### Olink technology

The Olink ^®^ Target 96 platform utilises PEA technology ^5, 7^ to measure proteins. This technology works by using two antibodies for each target protein which are linked to oligonucleotides. When both antibodies bind to the target protein, the barcoded oligonucleotides are able to hybridise providing a template for DNA polymerase and polymerase chain reaction (PCR) amplification. Quantitative PCR (qPCR) is used to quantify the protein concentration in normalised protein expression (NPX) units, which are arbitrary units on a log _2_ scale ^7, 16^. This method of detection is more sensitive than other methods such as enzyme-linked immunosorbent assays (ELISAs) ^17^.

The Olink ^®^ Target 96 Inflammation panel detects a set of 92 proteins with known involvement in inflammatory responses as determined by a combination of UniProt, Human Protein Atlas, Gene Ontology and DisGeNET ^18^. It is typically applied to samples on a 96-well plate, including 88 customer samples and 8 control samples. Proteins in the panel comprise a broad selection of proteins involved in a range of well-known biological processes including apoptotic processes, cellular responses to cytokine stimuli and the inflammatory response. This array of inflammatory proteins therefore provides a detailed readout of an individual’s inflammatory status. A full list of the proteins and their UniprotID measured using this panel is provided in Supplementary Table 1 in the Extended data.

### Data as provided by Olink

The heparin plasma samples were analysed on 104 plates in 7 batches using the Olink® Target 96 inflammation panel. Each plate included samples from each of the three groups (mothers, 9-year-olds and 24-year-olds). What follows is a description of the steps and analyses that Olink Proteomics performed for this data release. Four internal controls were added to each sample (or well) to monitor the quality of assay performance, as well as the quality of individual samples. The quality control (QC) was performed in two steps: 1) Each sample plate was evaluated on the standard deviation of the internal controls. This should be below 0.2 NPX. Only data from sample plates that pass this quality control are reported. 2) The quality of each sample was assessed by evaluating the deviation from the median value of the controls for each individual sample. Samples that deviate less than 0.3 NPX from the median pass the quality control. Data from all samples were included in the data output file provided to us by Olink. Samples that did not pass the QC were indicated in columns named "QCWarning". Data points from samples that did not pass QC should be treated with caution. 8903 (99%) of the samples passed Olink QC. Intra- and inter-assay coefficients of variance (%CV) were calculated by Olink, based on control samples (pooled plasma samples) included on each plate. The average intra-assay CV was 7% and the average inter-assay CV was 15%. Based on protein detection information returned by Olink, 66 out of the 92 proteins (72%) were detected in >75% of the samples. Data values for measurements under the level of detection (LOD) were reported for all samples and retained in the dataset as it could be biologically relevant that there is data <LOD. The LOD values and the percentage of samples <LOD are provided for each of the 92 proteins in Supplementary Table 1 in the Extended data. The Olink Certificate of Analysis is also included in the Extended data. Data were returned to ALSPAC as normalised protein expression (NPX) values, Olink Proteomics’ arbitrary unit on log _2_ scale. Fifteen of the 9000 samples failed Olink QC and consequently have missing data for all 92 proteins.

### Data summary and pre-analytical processing

Proteomics data were summarised and further QC filters applied with a view to delivering a high-quality dataset for future statistical analysis. This was done using *metaboprep* ^19^, an R package that provides a standardised data processing workflow to extract data from pre-formed worksheets, generate summary statistics and support sample and feature (here, proteins) filtering by a variety of quality metrics. After summary statistics were calculated on the raw data, which included data values <LOD, a series of QC filters were applied. As each plate included samples from the mothers, 9-year-olds and 24-year-olds, we analysed all 9000 samples together with *metaboprep*. First, samples with ≥80% missing data (across the 92 proteins) were excluded and then proteins with ≥80% missing data (across the 9000 samples) were excluded. These first two filtering parameters remove samples and proteins with extreme missingness. After the filtering of extremes, missingness was re-estimated. Next samples were filtered on total sum abundance (TSA) estimates, removing those that were five interquartile range unit distances from the sample population median TSA. The TSA summary statistic estimates the total signal (or abundance) across all complete proteins (proteins that have no missingness in the sample population) within each sample and helps identify sample outliers influenced by sample dilution or other broadly influencing technical error(s). Principal components (PC) for samples were then estimated using representative proteins, that is those identified to be statistically independent, and setting the parameter “outlier_treatment” to “leave_be” which, for each sample, takes no action on outlying protein values. The representative proteins were identified by generating a hierarchical clustering dendrogram based on Spearman’s rho distances and performing a tree cut at the height of 0.5, corresponding to a Spearman’s rho of 0.5. As such, for each cluster of proteins with a Spearman’s rho greater than or equal to a value of 0.5 a single principal variable was identified and carried forward as the representative protein. Those proteins not in a cluster were their own representative protein and were carried forward into the PC analysis. Samples in the first N PCs that were greater than five standard deviations from the mean were removed, where N is the number of PCs determined by the Cattel’s Scree Test acceleration factor. Finally, the last step of the *metaboprep* pipeline was to generate, for each protein, a scatter plot identifying outlying data points, a histogram of the same values and a table that includes selected summary statistics. We have included this, along with the summary report and log file in the Extended data. The sample indexes on the scatterplots for each protein are indexed in the order of mothers, 24-year-olds and 9-year-olds.

Using the *metaboprep* filtered dataset (performed on the whole sample), we summarised subject characteristic data on age, sex, body mass index (BMI), ethnicity, smoking status and socioeconomic variables, for which we used education beyond A-levels (Advanced level qualification - optional examinations taken at the approximate age of 18) and manual occupation, for the sample as a whole and (where relevant) for each of the three age groups. Information was obtained from clinics/questionnaires at the same/similar timepoint as the blood samples were taken ( https://www.bristol.ac.uk/alspac/researchers/our-data/questionnaires/). Current smoker status was obtained by questionnaire for the mothers (T) and from the Focus@24 clinic for the 24-year-olds. For the 9-year samples we used information whether anyone in the household smoked to estimate if they were exposed to smoke (Q). We also calculated correlation matrices between the 92 Olink proteins on the filtered dataset.

As a sensitivity analysis, we ran *metaboprep* for each age group separately to identify how many additional outlier samples there would be in this case, but these datasets were not used in this paper and will not be generally available from ALSPAC.

### Validation of platform

Interleukin-6 (IL-6) was previously assessed in the samples from the Focus@9 clinic by clinical chemistry measurements using enzyme-linked immunosorbent assay (R&D Systems, Abingdon, UK) ^20^. We calculated the Pearson’s r correlation coefficient between these IL-6 measurements and the Olink IL-6 measurements from the same samples. 38% of all Olink IL-6 data values <LOD, but this data was retained in the dataset as it was considered to be biologically relevant. As a sensitivity analysis, we calculated the correlation coefficient on the subsample with Olink IL-6 >LOD.

As a positive control analysis, we used a previously well described and biologically understood association ^21^ – that between IL-6 and adiposity – to illustrate the validity and utility of this data release. To do so we estimated Pearson’s r correlation coefficients between BMI and Olink measured IL-6 in each age group, separately. We used the *metaboprep* filtered dataset applied to the whole sample and then split this dataset by the three age groups. IL-6 measured by clinical chemistry in the Focus@9 samples (9-year-olds) was also include in this analysis. As a sensitivity analysis, we calculated the correlation coefficients on the subsamples with Olink IL-6 >LOD.

We also estimated the correlations between all Olink proteins with other inflammatory biomarkers previously measured in ALSPAC - namely C-reactive protein (CRP) and glycoprotein acetyls (GlycA). This was not a validation exercise, but an investigation into whether any of the Olink proteins could be used as a proxy for CRP and/or GlycA. CRP was measured by automated particle-enhanced immunoturbidimetric assays supplied by Roche (Indiana, USA) ^22^. GlycA was assayed as a part of the metabolomics profiling performed on the Nightingale® Health NMR metabolomics platform (Helsinki, Finland) using blood plasma samples ^23– 25^. Mothers from the FOM1 clinic and the 24-year-olds from the Focus@24 clinic were run on this platform, but samples from the Focus@9 clinic were not, so we used data from the nearest timepoint available that was, namely the Focus@7 clinic where the children were approximately 7 years old. For both assays, mothers (FOM1) and young adults (Focus@24) had fasted, whereas the children (Focus@9 and Focus@7) had not.

## Results

### Pre-analytical processing by *metaboprep*

**Figure 3.**
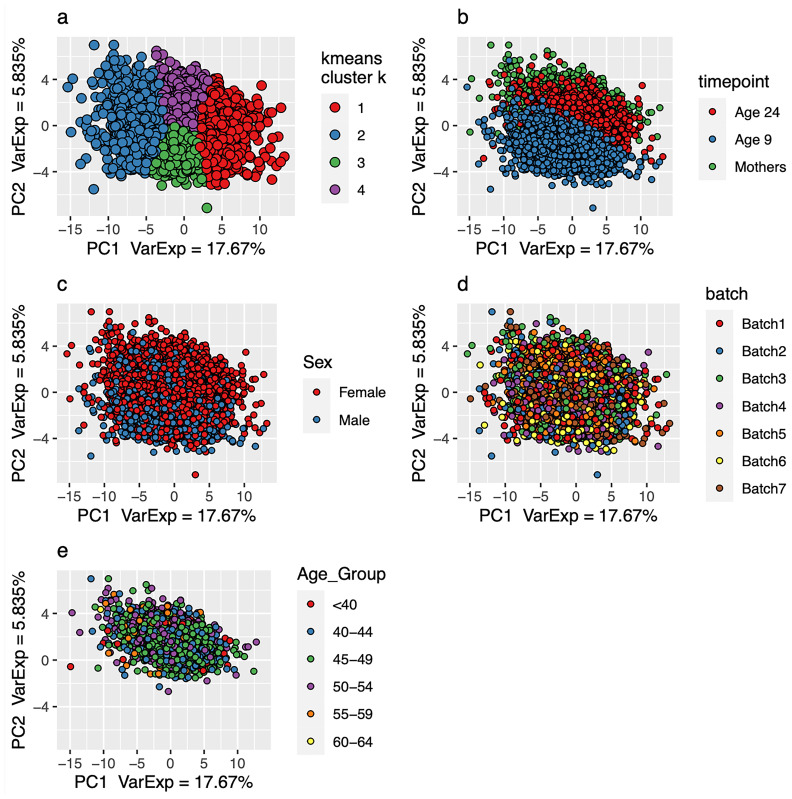
Plot of principal component 1 and 2 derived from 63 representative proteins in 8983 samples. Samples included passed *metaboprep* QC. ( **a**) Individuals in the principal component (PC) plot were clustered into 4 k-means (k) clusters, using data from PC1 and PC2. The k-means clustering and colour coding is strictly to help provide some visualisation of the major axes of variation in the sample population(s). Individuals in the PC plot are colour-coded by timepoint ( **b**), sex ( **c**), batch ( **d**) and age group (mothers only ( **c**)).

**Figure 4.**
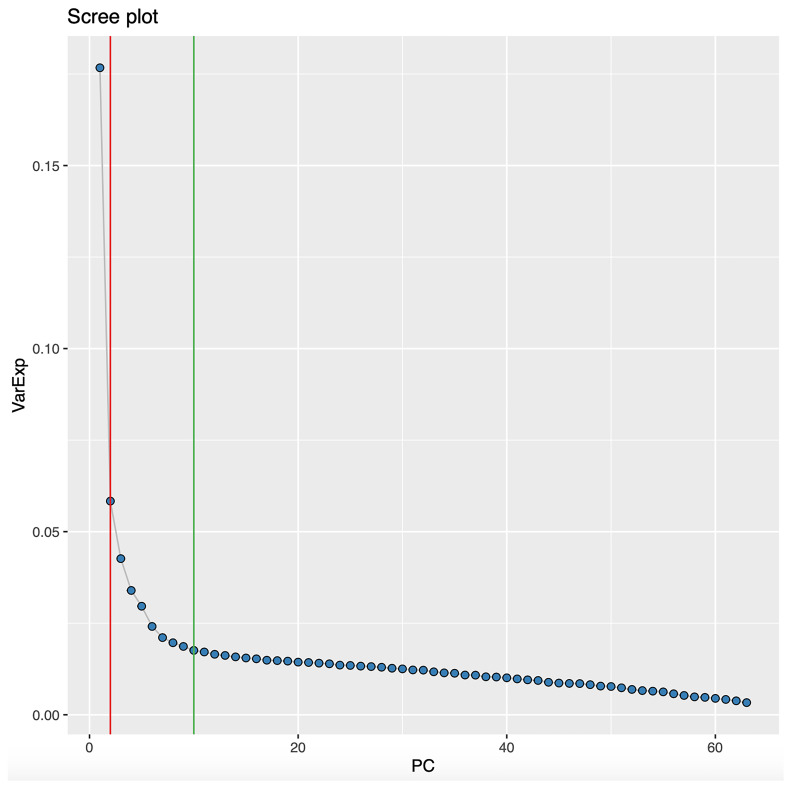
Scree plot of the variance explained by each principal component. Derived from the 63 representative proteins in 8983 samples, the Scree plot also identifies the number of principal components estimated to be informative (vertical lines) by the Cattel's Scree Test acceleration factor (red, n = 2) and Parallel Analysis (green, n = 10).

**Figure 5.**
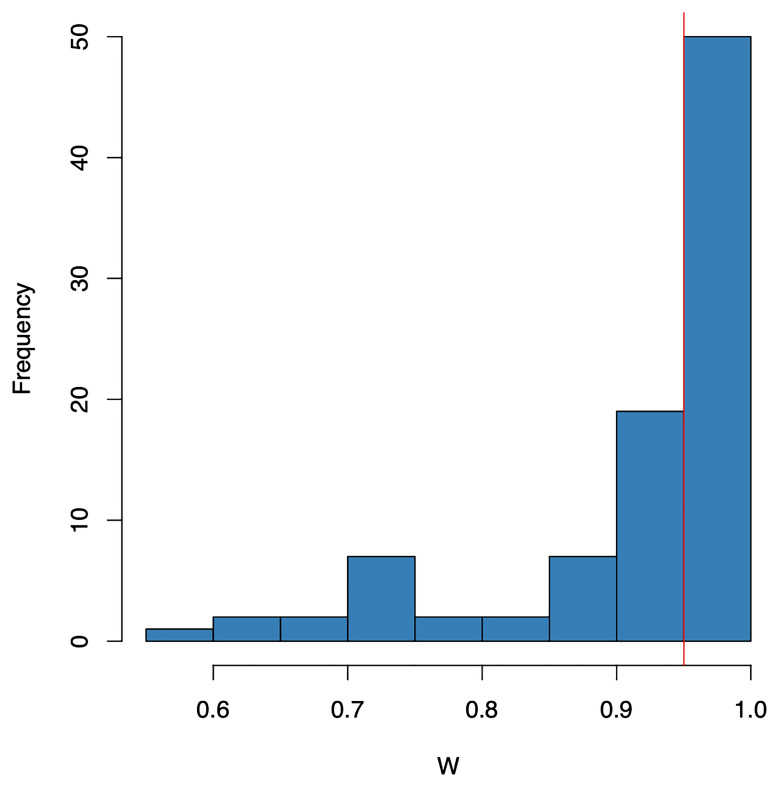
Histogram of the Shapiro-Wilk W-statistics for each Olink protein (N=92). A W-statistic value of 1 indicates the sample distribution is perfectly normal and value of 0 indicates it is perfectly uniform. The red vertical bar denotes a W statistic of 0.95.

**Figure 6.**
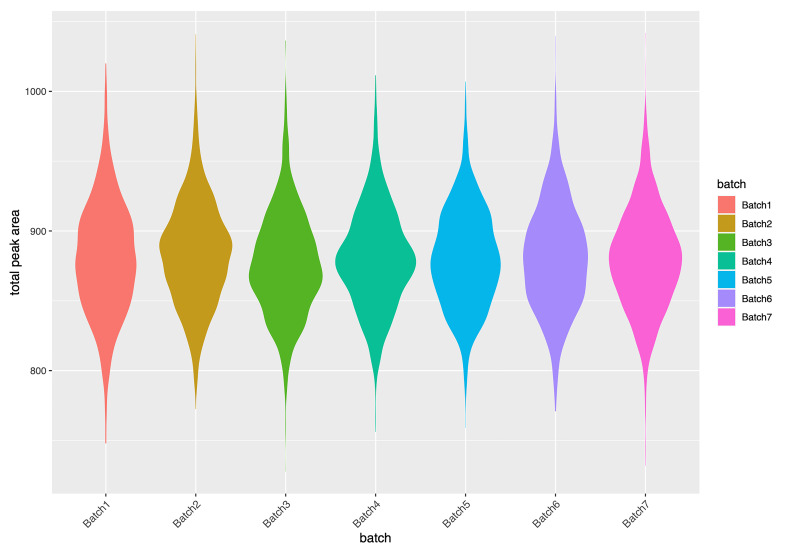
Violin plot illustrating the relationship between total sum abundance (TSA) and sample batch numbers.

In addition to the 15 samples that were removed by Olink QC procedures, two additional samples were detected as outliers by *metaboprep* (TSA/PCA values) and removed, leaving 8983 samples in the filtered dataset ( Figure 2), consisting of 2962 mothers, 3000 children and 3021 young adults. Across the 8983 mother and children/young person samples 2282 mothers have a child analysed at 9 years of age, 1630 at 24 years of age, and 1112 at both 9 and 24 years of age. In addition, 1829 of the children were analysed at both timepoints (9 and 24 years of age; see Figure 1b). Using the Spearman’s correlation distance tree cutting method with a Spearman’s rho of 0.5, a total of 63 representative features (proteins) were identified. The first two principal components of the protein measurements do not show any obvious evidence of sample clustering ( Figure 3a). We have also colour-coded the first two principal components by timepoint (mothers, 9-year-olds and 24-year-olds – Figure 3b), sex ( Figure 3c), batch ( Figure 3d) and age group (mothers only – Figure 3e). Cattel's Scree Test acceleration factor indicates 2 informative principal components and Parallel Analysis indicates 10 ( Figure 4). Out of the 92 proteins, 50 had near-normal distributions (Shapiro’s W statistic >0.95; Figure 5). There was no evidence of batch effects as seen in Figure 6 which provides an illustrative evaluation of the total abundance (at complete features) as a product of sample batch numbers. In total just 0.33% of the variation in TSA can be explained by batch number ( Figure 6). This filtered dataset of 8983 samples is available in the ALSPAC data in the original Olink NPX units, split into separate datasets for the mothers and offspring. The data was released by the ALSPAC data team in release version 6a in February 2022 (see data dictionary). These datasets will also include plate ID and Olink QC warning. Protein metadata will also be made available in ALSPAC, including assay, Uniprot ID, LOD and missing frequency. Supplementary Table 1 in the Extended data provides the ALSPAC variable names for each of the 92 proteins at each of the three timepoints. From the samples that passed *metaboprep* QC, 83 (0.9%) received a QC warning from Olink.

We also ran *metaboprep* for each age group separately. In doing so, when compared to running *metaboprep* on the whole sample, an extra three mothers, four 9-year-olds and three 24-year-olds were removed due to being PCA outliers/exceeding the TSA threshold. The log files are included in the Extended data. This was performed solely to identify how many extra outliers there would be by performing *metaboprep* on each of the three age groups separately and these three separate datasets are not subsequently used in this paper for validation or positive control analyses.

### Characteristics of ALSPAC participants with Olink data

Table 1 gives an overview of the subject characteristics for the 8983 samples that passed the *metaboprep* quality control performed on the whole sample, which consists of 2962 mothers, 3000 children and 3021 young adults. Subject characteristics are provided for the whole sample (where appropriate) and for each of the three age groups separately.

### Correlation between Olink inflammatory proteins

**Figure 7.**
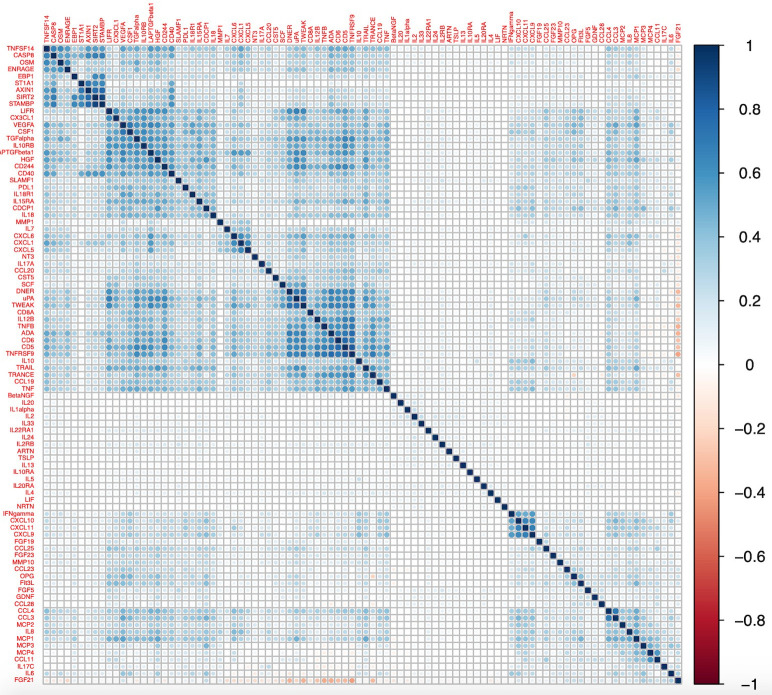
Heatmap of correlation matrix for 92 Olink proteins from all participants (N= 8983). A hierarchical clustering order in the R package ‘corrplot’ was used.

A heatmap depicting the correlation matrix for this dataset is presented in Figure 7, using a hierarchical clustering order. Pearson correlation estimates ranged from the strongest inverse correlation –0.40 between fibroblast growth factor 21 (FGF21) and tumor necrosis factor receptor superfamily member 9 (TNFSRF9) to the strongest positive correlation 0.91 observed between STAM-binding protein (STAMBP) and SIR2-like protein (SIRT2). Figure 7 provides some evidence of clusters of proteins that are strongly correlated. The correlation matrices were also calculated for each age group separately and are presented in the Extended Data (Figures S1–S3). Correlations were comparable across the different age-groups with similar clusters seen in each; the Pearson’s correlation estimates between the upper triangular matrices of each of the sub-groups were 0.91-0.95. Within each age-group the maximum correlation was also between STAMBP and SIRT2 (0.91-0.92).

### Validation of Olink data

**Figure 8.**
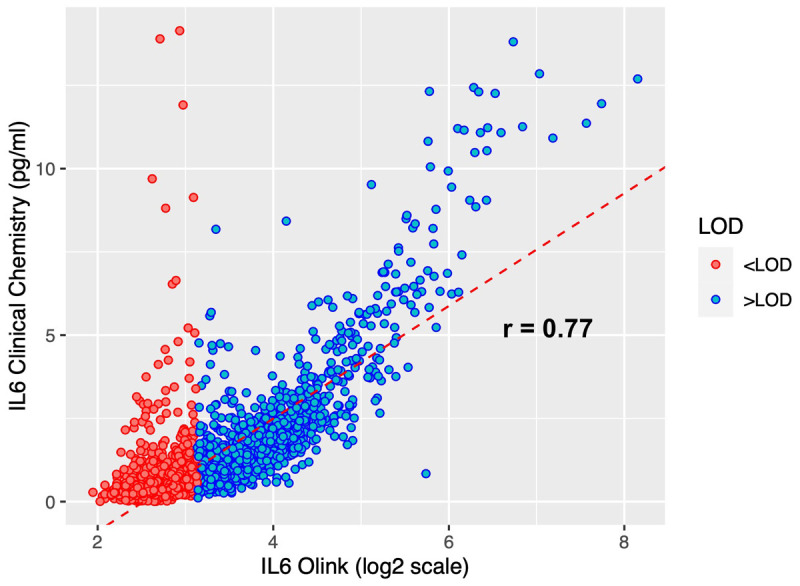
Scatter plot showing a comparison of Olink and clinical chemistry assay IL-6 measurements. Analyses are presented for N=2949 ALSPAC offspring at age 9. Olink measures IL-6 in log2 NPX units, whereas it is measured in picograms per millilitre (pg/ml) in the clinical chemistry assay. The red dotted line displays the univariable linear regression of IL-6 clinical chemistry on all log2 IL-6 Olink NPX data.

***Correlation between clinical and Olink measurements of IL-6*.** In total, 2949 samples had IL-6 assessed by clinical chemistry at 9 years of age. The Pearson correlation between these two different IL-6 measurements is 0.77 (95% CI 0.75-0.78), indicating consistency among the two technologies ( Figure 8). We repeated the correlation for the 1464 (49.6%) samples with Olink IL-6 >LOD, giving an estimate of r=0.87 (95% CI 0.85-0.88).

**Figure 9.**
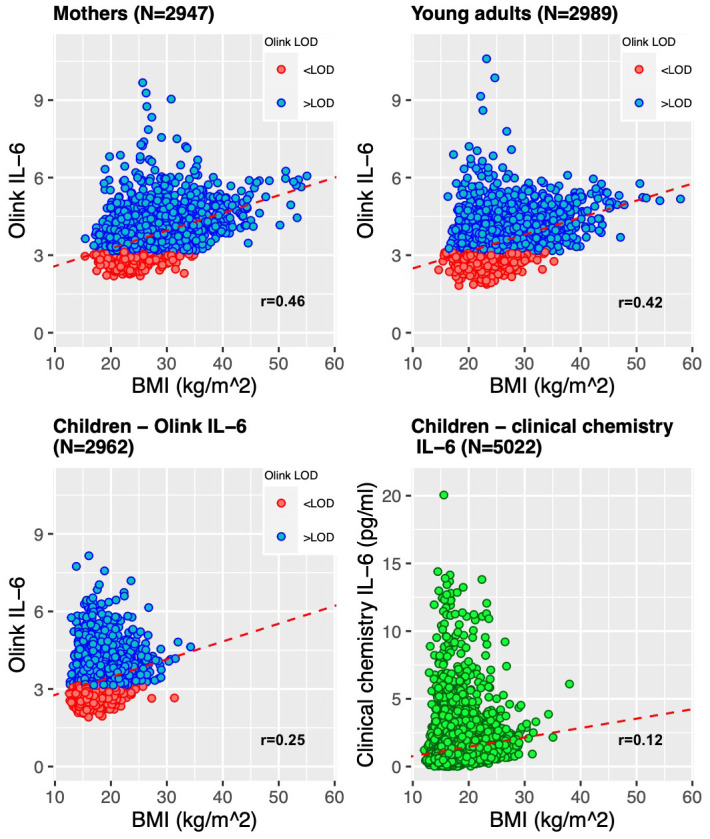
Scatter plots showing the comparison of BMI (body mass index) and Olink IL-6. Analyses are displayed for each age group – mothers, children (age 9) and young adults (age 24)) and IL-6 from children’s clinical chemistry assays, in picograms per millilitre (pg/ml). The red dotted lines represent the univariable linear regression of all log2 IL-6 Olink NPX data or IL-6 clinical chemistry on BMI data.

***Correlations between BMI and IL-6*.** Of the participants who have Olink data, nearly all (99%) have concurrent BMI data. Similarly, nearly all (99%) of the 9-year-olds who had IL-6 measured by clinical chemistry had concurrent BMI data from the same timepoint. We observed that IL-6 was associated with BMI in the entire Olink dataset and in each group, independently (P<0.05). The association increased as the age of the group increased. In 9-year-olds with IL-6 measured by Olink, mean IL-6 was raised 0.069 NPX per unit (kg/m ^2^) higher BMI (95%CI 0.059-0.078, r=0.25, p<0.001). This was a stronger correlation than observed with clinical chemistry IL-6 measurement. The slope was similar in 24-year-olds, but BMI displayed a stronger correlation with IL-6 (mean difference in IL-6 0.066 (95%CI 0.060-0.071) NPX per 1 kg/m ^2^ BMI, r=0.42, p<0.001). The strongest correlation was displayed in the mothers (mean difference in IL-6 0.068 (95%CI 0.064-0.073) NPX per 1 kg/m ^2^ higher BMI, r=0.46, p<0.001). The scatterplots for each sub-group are displayed in Figure 9.

As a sensitivity analysis, we estimated the associations using only Olink IL-6 values >LOD. The correlations were weaker in each of the reduced datasets. In the 2304 (78.2%) mothers with Olink IL-6 >LOD, mean IL-6 was raised 0.051 NPX per unit (kg/m ^2^) higher BMI (95%CI 0.046-0.056, r=0.39, p<0.001). In the 1817 (60.8%) 24-year-olds, mean IL-6 was raised 0.039 NPX per unit (kg/m ^2^) higher BMI (95%CI 0.034-0.045, r=0.30, p<0.001) and in the 1476 (49.8%) 9-year-olds, mean IL-6 was raised 0.021 NPX per unit (kg/m ^2^) higher BMI (95%CI 0.009-0.032, r=0.09, p<0.001).

**Figure 10.**
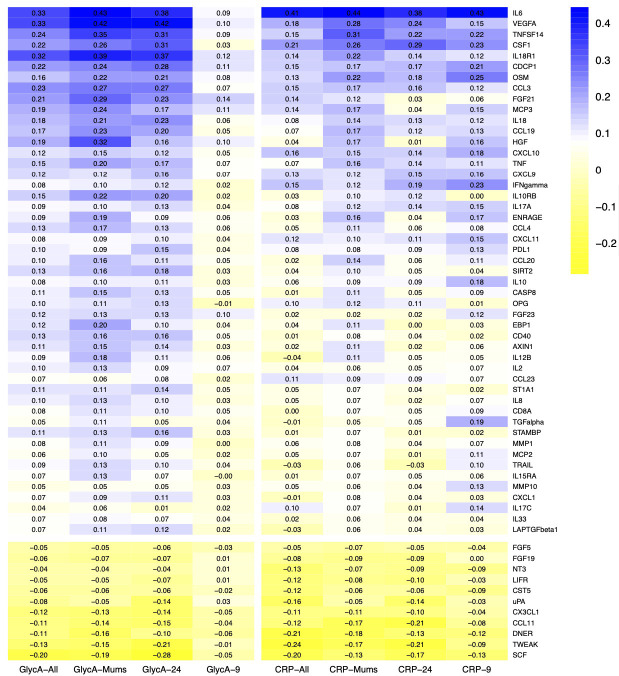
Heatmap of Pearson correlation estimates between GlycA and CRP and the Olink proteins. The analysis was performed using all participants and by segregating by age group. Only 60 out of the 92 Olink proteins are displayed, which all have an average absolute correlation >0.05.

***Correlation between CRP and GlycA and the Olink proteins*.** Pearson correlation estimates between CRP/GlycA and the Olink proteins are presented in Figure 10 for the whole sample and for each age group separately. Nearly all participants with Olink data (2874 (97%) mothers, 2953 (98%) 9-year-olds and 2806 (93%) 24-year-olds) also had CRP measurements above the limit of detection (LOD). IL-6 has the strongest correlation with CRP across all age groups (r = 0.37-0.44). There was a concern about the results from 24-year-olds as they had the highest percentage of CRP measurements below the LOD (7%). As a sensitivity analysis, we replaced these 209 values with the LOD value (0.1mg/L). This had virtually no effect on the correlation estimates, with a maximum difference <0.01 in the 24-year-olds.

Data on both Olink and GlycA were available for 2930 (99%) ALSPAC mothers from the FOM1 clinic and 2943 (97%) of the 24-year-olds. 2118 (71%) have both Olink at age 9 and GlycA measured at age 7. In the mothers and the 24-year-olds, the highest correlations were between GlycA and vascular endothelial growth factor A (VEGFA) (r=0.42, 0.42), IL-6 (r=0.43, 0.38), interleukin-18 receptor 1 (IL18R1) (r=0.39, 0.37) and TNFSF14 (0.35, 0.31). The correlation with hepatocyte growth factor (HGF) was higher in the mothers (r=0.32) than in the 24-year-olds (r=0.16). |r| < 0.33 for all other correlations in the mothers and 24-year-olds. The GlycA measured at age 7 was very weakly correlated with all of the Olink proteins at age 9 (|r| < 0.14).

For visualisation purposes, only 60 out of the 92 Olink proteins are displayed in Figure 10. All the other 32 proteins have an absolute value of the average correlation across all CRP and GlycA segregations <0.05.

## Discussion

In this data note we have described the Olink® Target 96 Inflammation panel performed in 9000 ALSPAC samples. We have provided details of proteomic data received from Olink, pre-analytical processing, participant characteristics and some analyses to validate the Olink data.

Given the importance of chronic inflammation as a risk factor, biomarker and potential cause of disease, coupled with the extensive health information collected by ALSPAC across the life course, analysis of this proteomic dataset will help us better understand the causes of chronic inflammation and its role in the development of non-communicable diseases. Novel findings are likely to clarify questions of timing given the longitudinal nature of ALSPAC and the fact that the proteomes have been measured in childhood, young adulthood and at mid-life, with a large proportion having proteomes measured at two time points.

*Metaboprep* was run on all 9000 samples at the same time to detect and filter outliers and the filtered dataset of 8983 samples is available in the ALSPAC data. To conform with the format of other ALSPAC ‘omics data (e.g. metabolomics), ALSPAC will split this dataset into 2 separate datasets, one for the G0 mothers and one for the G1 offspring. Only a small number of outliers were identified and removed by *metaboprep*. 83 (0.9%) of the samples that passed *metaboprep* QC received a QC warning from Olink; this is possibly due to more liberal thresholds used in *metaboprep* than Olink. We have kept the Olink QC column in the final dataset, named ‘Olink_QCWarning’, so that users can decide whether to use these samples. We observed that *metaboprep* identifies a small number of extra outliers if each age group is evaluated separately (three extra mothers, four extra 9-year-olds and three extra 24-year-olds). These extra outliers are not highlighted within the ALSPAC data, since the main focus was to run *metaboprep* on the entire experimental sample set of mothers, 9-year-olds and 24-year-olds. 

Our validation work suggests that IL6 measurements by Olink provide a meaningful readout of inflammation. This was supported by our validation analyses. For example, BMI and IL-6 have been shown to be positively associated in observational studies ^26^. We replicated this positive correlation between BMI and IL-6 in all subgroups. Estimates from linear regression in the adult subgroups (24-year-olds and the mothers) were comparable to published estimates in adults, however the slope of linear model and correlation coefficients indicated the relationship was slightly stronger than published estimates ^26– 28^. These associations were weaker when analyses were restricted to the Olink IL-6 values >LOD. The correlation was stronger when using the IL-6 measurement by Olink compared to the clinical chemistry IL-6 measurement in the Focus@9 children. As well as this, measurements of CRP and GlycA displayed correlations with other inflammatory proteins, but none of the Olink proteins were correlated strongly enough to be regarded as a proxy for CRP and/or GlycA. When comparing IL-6 measurements from Olink and clinical chemistry, the shape of the data ( Figure 8) is indicative of different sensitivities at the upper end. While validation of the IL6 abundance is positive, it cannot be used as a validation of the whole Olink panel, as each protein is detected by a unique set of antibodies, and the specificity may vary across proteins.

As GlycA data was not available at age 9, we used data from age 7 to correlate with the 9-year-olds’ Olink protein levels. However, since none of the Olink proteins were correlated with the GlycA at age 7, then it appears that GlycA at age 7 may not be representative of GlycA at age 9.

## Strengths and limitations

### Strengths

ALSPAC is a longitudinal cohort study that has collected data on the parents prior to the birth of the index child and followed them up regularly ever since. The strengths of the ALSPAC data include the large sample size of participants (women, partners and offspring) with data available ^8^. The only requirement for inclusion at enrolment was the geographical location in which the mother resided and the expected date of delivery (April 1991-Dec 1992). The participants recruited to the study were broadly representative of the general population of new parents' resident in the area at the time in terms of sex, ethnicity and socio-economic status ^9^. A significant strength of this study is that we have been able to obtain inter-generational proteomic data from mothers and their offspring, with a large family overlap. Furthermore, the data that will be released by ALSPAC will have undergone two separate quality control analyses.

### Limitations

As with all longitudinal studies, attrition is a problem over time. It has been higher for those who experienced greater adversity during enrolment and the index pregnancy (e.g. inadequate housing and lack of social support). Latterly, for these study parents, the loss is due to several reasons, including change of address and consequent loss to follow up, mortality and reluctance to stay involved in the study. This skews the current participants to those less socioeconomically deprived and with higher educational attainment. However, linkage and data are still available for participants who have left the study, unless they have requested for it to be removed. A further limitation of the study is the lack of ethnic diversity. At the time of enrolment, the county of Avon was mainly white, therefore there were too few non-white participants (<6%) to allow for detailed analysis by ethnic background. Despite these limitations, for a study of this length to be able to retain about 50% of the original participants is still excellent, with a good representation of the pregnancies which accounted for approximately 80% of the population within the sampling frame and from which future generations have been followed within ALSPAC.

There are some limitations of this dataset that users should consider. Firstly, Olink provided protein detection results, which was the number of proteins detected and with measures above the LOD in >75% of the samples. 66 out of the 92 proteins (72%) were detected in >75% of the samples. Olink’s expected detectability in EDTA plasma is >75%, which is based on EDTA plasma from healthy donors. Olink report that values are intended as guidelines only and protein levels may vary depending on different pathological conditions, sample matrices, or sample preparation methods. It is not clear why these samples did not reach 75% detectability. Our blood samples were taken into vacutainers containing heparin. It is possible that this choice in anticoagulant could have reduced the protein detectability. We retained the data values <LOD in the dataset as they were regarded as biologically relevant; this includes IL-6 (38% samples <LOD) which we used in validation exercises. The LODs for each protein and the % missingness due to <LOD will also be released by the ALSPAC data team, so that researchers can decide whether to include proteins with a large proportion of data values <LOD.

Despite the pre-processing of the data, this data has not undergone any transformations. Although the Shapiro-Wilk test indicated that levels of 42 proteins were not normally distributed (in their original NPX units), very few proteins were extremely non-normal. However, users of this data need to be aware that data transformations may be necessary for certain analyses. A further limitation is that samples at age nine were from non-fasted participants but samples at age 24 were from participants who fasted, limiting the direct comparison of protein abundances for participants who were measured at both timepoints. There appears to be a timepoint effect for some of the proteins (see Figure 3b and Extended data), which could potentially be due to age and/or possibly even time in freezer and non-fasted versus fasted. Users should consider whether appropriate to adjust for these variables in their analyses.

## Conclusion

Overall, we have provided a summary of the Olink® Target 96 Inflammation panel and details of the pre-processing of the 9000 samples from the ALSPAC cohort. We have also provided some reassuring validation analyses, suggesting that the panel is able to capture information about the participants’ inflammatory status. The filtered dataset of 8983 samples available in the ALSPAC data is therefore a rich data source to enable researchers to explore the role of inflammation in a vast range of diseases. There is a considerable family overlap; across the 8983 mother and children/young person samples 2282 mothers have a child analysed at 9 years of age, 1630 at 24 years of age, and 1112 at both 9 and 24 years of age. In addition, 1829 of the children were analysed at both timepoints (9 and 24 years of age).

## Data Availability

ALSPAC data access is through a system of managed open access. The steps below highlight how to apply for access to the data included in this data note and all other ALSPAC data. The datasets presented in this article are linked to ALSPAC project number B3821, please quote this project number during your application. The ALSPAC variable codes highlighted in the dataset descriptions can be used to specify required variables. 1. Please read the ALSPAC access policy ( www.bristol.ac.uk/media-library/sites/alspac/documents/researchers/data-access/ALSPAC_Access_Policy.pdf) which describes the process of accessing the data and samples in detail, and outlines the costs associated with doing so. 2. You may also find it useful to browse our fully searchable research proposals database ( https://proposals.epi.bristol.ac.uk/?q=proposalSummaries), which lists all research projects that have been approved since April 2011. 3. Please submit your research proposal ( https://proposals.epi.bristol.ac.uk/) for consideration by the ALSPAC Executive Committee. You will receive a response within 10 working days to advise you whether your proposal has been approved. If you have any questions about accessing data, please email alspac-data@bristol.ac.uk. Open Science Framework: Supplementary information supporting this submission can be found on the Open Science Framework “Inflammation proteomics datasets in the ALSPAC cohort” project page, https://doi.org/10.17605/OSF.IO/AS95U ^29^. This project contains the following extended data: ‘Supplementary Information.docx’ (containing the log file for *metaboprep* run on the whole sample, correlation matrices for the 92 proteins from each age-group and the log file for *metaboprep* run on each age-group separately) ‘Supplementary_Table1.csv’ (including protein metadata such as full name, UniprotID, LOD, %<LOD and the ALSPAC variable names for each protein at each timepoint) *metaboprep* summary report ‘Feature distributions.pdf’ (containing, for each protein, a scatter plot identifying outlying data points, a histogram of the same values and a table that includes selected summary statistics) Olink certificate of analysis Data are available under the terms of the Creative Commons Attribution 4.0 International license (CC-BY 4.0).
