# Supplementary material for: Inflammation proteomics datasets in the ALSPAC cohort
Source: Wellcome Open Res. 2024 Feb 6;7:277. Originally published 2022 Nov 9. [Version 2] doi: 10.12688/wellcomeopenres.18482.2 (PMC11391261; doi:10.12688/wellcomeopenres.18482.2)
Supplement: Peer review history

# Reviewer report: Reviewer response for version 2

**DOI:** 10.21956/wellcomeopenres.23228.r73943

**Published:** 2024-02-19

**Authors:** Cecilia Engel Thomas

**Affiliations:** 1 University of Copenhagen, Copenhagen, Denmark

The authors have addressed the comments in my initial peer review report appropriately and I have no further feedback.

Are sufficient details of methods and materials provided to allow replication by others?

Yes

Is the rationale for creating the dataset(s) clearly described?

Yes

Are the datasets clearly presented in a useable and accessible format?

Partly

Are the protocols appropriate and is the work technically sound?

Yes

Reviewer Expertise:

Antibody based proteomics, bioinformatics

I confirm that I have read this submission and believe that I have an appropriate level of expertise to confirm that it is of an acceptable scientific standard.

# Reviewer report: Reviewer response for version 1

**DOI:** 10.21956/wellcomeopenres.20496.r61268

**Published:** 2023-07-10

**Authors:** Cecilia Engel Thomas

**Affiliations:** 1 University of Copenhagen, Copenhagen, Denmark

In this data note the authors present data from the profiling of 96 proteins using Olink in 9,000 plasma samples from the ALSPAC study. Samples originate from three study groups: mothers, offspring at age 9 and offspring at age 24. The data have been quality controlled by Olink using their standard pipeline and additionally the authors have quality controlled the data using the *metaboprep* R package. The authors compare the levels of IL6 measured by Olink and clinical chemistry and find a high correlation. Additionally, association of IL6 with body mass index, shows a positive correlation in line with previous findings. In summary, the presented data is a valuable resource for studying the profiled proteins at different life stages across many individuals, and the linkage to the rich phenotypic data available in ALSPAC is a big strength of the study.

 QC steps are well documented, both in the manuscript text and in the supplementary material.

 The authors use the R package *metaboprep* for assessing the quality of the Olink data. This package is developed for metabolomics data and is not commonly used for analysis of Olink data. Therefore, it would be helpful if the authors could comment on to which extent the methods implemented in the package are appropriate for Olink data.

 While there are no differences observed in the overall protein abundance or missingness per batch (as seen in the *metaboprep* report) there seems to be a batch effect at the individual protein level as seen from the feature distribution plots (“Feature distributions.pdf”). A large number of proteins (e.g. ADA, CCL19, CCL20, CD5, CD6) have a distinctly higher level for a subset of the ~2,600 individuals with the highest sample indexes, while other proteins (e.g. CCL28, CDCP1) have a distinctly higher level for the ~2,600 individuals with the lowest sample indexes. It would be helpful for future users of the data if the authors could highlight this in the manuscript and comment on whether this is indeed a batch effect or an effect of the three study groups (FOM1, Focus@9, Focus@24).

 Figure 3 shows that there is no clear structure to the data. However, principal components in the data may still be driven by covariates such as age and sex, and preanalytical variables such as study center (if multiple were involved) and time-in-freezer/sampling date. It would be helpful for future users of the data if the authors provided some basic insights into which of these variables may be necessary to consider in future analyses. This could e.g. be done simply by coloring the PC plot by these variables or more stringently by associating these variables to PCs or individual proteins.

 The authors run the *metaboprep* pipeline on each age group separately and find additional outliers, but ultimately these individuals are kept in the data. Do the authors recommend that these samples be included or excluded? Further, it will be relevant to highlight these individuals in any subject meta data provided.

 The authors highlight the longitudinal nature of the data as a strength of the study. However, in the instances where multiple samples were taken from the same individual one sample was non-fasted and the other fasted, limiting the direct comparison of protein abundances at these time points. I suggest that the authors highlight this as a limitation in the discussion.

 While validation of the IL6 abundance is positive, it cannot be used as a validation of the whole Olink panel, as each protein is detected by a unique set of antibodies, and the specificity may vary across proteins. I suggest the authors clarify this further when discussing the validation steps.

 I was not able to assess whether the datasets were clearly presented in a useable and accessible format since accessing the data (subject meta data and protein measurements) requires submitting a research proposal. However, the protein meta data provided as part of the manuscript appeared sufficient and was clearly described.

 I have no expertise within immunology and can therefore not comment on those aspects of the manuscript.

 Minor comments:

 - There are several figures in the main text with low resolution. Figures 3, 4, 5 and 6.

Are sufficient details of methods and materials provided to allow replication by others?

Yes

Is the rationale for creating the dataset(s) clearly described?

Yes

Are the datasets clearly presented in a useable and accessible format?

Partly

Are the protocols appropriate and is the work technically sound?

Yes

Reviewer Expertise:

Antibody based proteomics, bioinformatics

I confirm that I have read this submission and believe that I have an appropriate level of expertise to confirm that it is of an acceptable scientific standard, however I have significant reservations, as outlined above.

# Response

**Published:** 2023-12-18

**Authors:** Neil Goulding

**Affiliations:** University of Bristol, UK

*We thank the reviewer for their comments. We have responded to the reviewer’s comments in turn below, with the reviewer’s original comments in standard font and our responses in italics.*

 The authors use the R package metaboprep for assessing the quality of the Olink data. This package is developed for metabolomics data and is not commonly used for analysis of Olink data. Therefore, it would be helpful if the authors could comment on to which extent the methods implemented in the package are appropriate for Olink data. 

*Although metaboprep has been mainly used for metabolomics data, the package can also be used for proteomic data, as long as the input data is in the correct format. All parts of the pipeline (e.g. exploring missingness, performing a principal component analysis, identifying outliers, exploring distributions of the data and exploring the structure within the data) are as relevant for proteomic data as they are for metabolomic data. The user has control over setting parameters, such as outlier thresholds and tree-cutting heights, within the parameter file, therefore, these can be altered to ensure the suitability to the proteomic data.*

 While there are no differences observed in the overall protein abundance or missingness per batch (as seen in the *metaboprep *report) there seems to be a batch effect at the individual protein level as seen from the feature distribution plots (“Feature distributions.pdf”). A large number of proteins (e.g. ADA, CCL19, CCL20, CD5, CD6) have a distinctly higher level for a subset of the ~2,600 individuals with the highest sample indexes, while other proteins (e.g. CCL28, CDCP1) have a distinctly higher level for the ~2,600 individuals with the lowest sample indexes. It would be helpful for future users of the data if the authors could highlight this in the manuscript and comment on whether this is indeed a batch effect or an effect of the three study groups (FOM1, Focus@9, Focus@24). *Thank you for raising this point. This is an effect of the three study groups; the first 2968 sample indexes refer to the mothers, the next 3027 indexes refer to the 24-year-olds and the final 3005 indexes refer to the 9-year-olds. We agree that this needs highlighting in the manuscript and we have added the following to the end of the first paragraph of the Methods subsection ‘Data summary and pre-analytical processing’:*

*“The sample indexes on the scatterplots for each protein are indexed in the order of mothers, 24-year-olds and 9-year-olds.”*

 Figure 3 shows that there is no clear structure to the data. However, principal components in the data may still be driven by covariates such as age and sex, and preanalytical variables such as study center (if multiple were involved) and time-in-freezer/sampling date. It would be helpful for future users of the data if the authors provided some basic insights into which of these variables may be necessary to consider in future analyses. This could e.g. be done simply by coloring the PC plot by these variables or more stringently by associating these variables to PCs or individual proteins. *We have re-made Figure 3 as a multiplot. It now includes the original Figure 3 as Figure 3a, plus 4 other PC subplots, colour-coded by timepoint (mothers, 9-year-olds and 24-year-olds), sex and batch. We have also included a subplot (Figure 3e) which colour-codes the PC plot by age group (for the mothers only). We have not estimated the association between these variables and PCs or individual proteins because it is beyond the scope of this data note. We have edited one sentence and added a new sentence in the first paragraph of the Results section:*

*“* *The first two principal components of the protein measurements do not show any obvious evidence of sample clustering (Figure 3a). We have also colour-coded the first two principal components by timepoint (mothers, 9-year-olds and 24-year-olds – Figure 3b), sex (Figure 3c), batch (Figure 3d) and age group (mothers only – Figure 3e).”* *Viewing the new Figure 3b and from your above comment on the protein distributions, it is evident that there is a timepoint effect for some of the proteins. We believe that this is probably due to age but could also potentially be due to other factors such as time in freezer and fasted versus non-fasted. However, we do not see consistently lower levels in the 9-year-olds (whose samples have been frozen for the longest) and there is no evidence of increased noise in the feature distributions. We have added the following sentence to the end of the Discussion:*

*“There appears to be a timepoint effect for some of the proteins (see Figure 3b and Extended data), which could potentially be due to age and/or possibly even time in freezer and non-fasted versus fasted. Users should consider whether appropriate to adjust for these variables in their analyses.”* *We have also changed the title and footnote for Figure 3:*

*“ **Figure 3: P*** ***lot of principal component 1 and 2 derived from 63 representative proteins in 8983 samples.*** *Samples included passed metaboprep QC. (a) Individuals in the principal component (PC) plot were clustered into 4 k-means (k) clusters, using data from PC1 and PC2. The k-means clustering and colour coding is strictly to help provide some visualisation of the major axes of variation in the sample population(s). Individuals in the PC plot are colour-coded by timepoint (b), sex (c), batch (d) and age group (mothers only (c)).”*

 The authors run the metaboprep pipeline on each age group separately and find additional outliers, but ultimately these individuals are kept in the data. Do the authors recommend that these samples be included or excluded? Further, it will be relevant to highlight these individuals in any subject meta data provided. *The main focus was to run the metaboprep pipeline on the entire sample of mothers, 9-year-olds and 24-year-olds because all of the plates that were analysed by Olink included samples from each of the three groups, which is explained in the data note. We consider all these samples as a single experiment and therefore the data should be processed in this way. As we point out in the final paragraph of the Methods subsection ‘Data summary and pre-analytical processing’, we only ran metaboprep on each group separately as a sensitivity analysis to see if many extra outliers were highlighted in this way. Very few extra outliers were found by running it separately and we suggest that these data points should remain in the main dataset. If researchers wanted to, they could derive lists of these extra outliers by running metaboprep themselves on the released data. We have added further clarification by adding the following sentence to the Discussion:* *“These extra outliers are not highlighted within the ALSPAC data, since the main focus was to run metaboprep on the entire experimental sample set of mothers, 9-year-olds and 24-year-olds.”*   The authors highlight the longitudinal nature of the data as a strength of the study. However, in the instances where multiple samples were taken from the same individual one sample was non-fasted and the other fasted, limiting the direct comparison of protein abundances at these time points. I suggest that the authors highlight this as a limitation in the discussion. *We agree with the Reviewer that having fasted and non-fasted data from the two offspring timepoints is a limitation. We have added the following sentence to the end of the Discussion:*

*“A further limitation is that samples at age nine were from non-fasted participants but samples at age 24 were from participants who fasted, limiting the direct comparison of protein abundances for participants who were measured at both timepoints.”*

 While validation of the IL6 abundance is positive, it cannot be used as a validation of the whole Olink panel, as each protein is detected by a unique set of antibodies, and the specificity may vary across proteins. I suggest the authors clarify this further when discussing the validation steps. *We agree with the point raised by the reviewer. We have edited the following sentence in the Discussion:* *“Our validation work suggests that protein measurements by Olink provide a meaningful readout of inflammation”,* *to replace the word ‘protein’ with ‘IL6’. We have also added the following sentence to the Discussion:*

*“While validation of the IL6 abundance is positive, it cannot be used as a validation of the whole Olink panel, as each protein is detected by a unique set of antibodies, and the specificity may vary across proteins.”*

 I was not able to assess whether the datasets were clearly presented in a useable and accessible format since accessing the data (subject meta data and protein measurements) requires submitting a research proposal. However, the protein meta data provided as part of the manuscript appeared sufficient and was clearly described.

*ALSPAC data access is through a system of managed open access.* *The ALSPAC variable codes highlighted in the dataset descriptions can be used to specify required variables (see data dictionary: https://www.bristol.ac.uk/alspac/researchers/our-data/ )* *. As mentioned in the Discussion, to conform with the format of other ALSPAC ‘omics data (e.g. metabolomics), ALSPAC have split the dataset into 2 separate datasets, one for the G0 mothers and one for the G1 offspring.*

 I have no expertise within immunology and can therefore not comment on those aspects of the manuscript.

 Minor comments:

 - There are several figures in the main text with low resolution. Figures 3, 4, 5 and 6. *We have re-made these figures with improved resolution.*

# Reviewer report: Reviewer response for version 1

**DOI:** 10.21956/wellcomeopenres.20496.r61276

**Published:** 2023-07-10

**Authors:** Melanie Föcking

**Affiliations:** 1 Royal College of Surgeons, Dublin, Leinster, Ireland

This is a very detailed and well written data note on ALSPAC samples on almost 9000 samples, including mothers, age 9 and age 24 offspring. The OLINK proteomic platform was used for the experimental part and data obtained is of great quality, verified by comparison to IL-6 in OLINK and single-plex ELISA from a previous experiment. The data will be available on request from ALSPAC for analysis by researchers. Therefore, results are not the main focus of this paper. The data note is very well executed and I cannot find any flaws.

Are sufficient details of methods and materials provided to allow replication by others?

Yes

Is the rationale for creating the dataset(s) clearly described?

Yes

Are the datasets clearly presented in a useable and accessible format?

Yes

Are the protocols appropriate and is the work technically sound?

Yes

Reviewer Expertise:

Proteomics

I confirm that I have read this submission and believe that I have an appropriate level of expertise to confirm that it is of an acceptable scientific standard.

# Response

**Published:** 2023-12-18

**Authors:** Neil Goulding

**Affiliations:** University of Bristol, UK

*We thank the reviewer for their positive review and comments.*
